# Factors associated with posttraumatic growth among North Korean defectors in South Korea

**DOI:** 10.1038/s41598-022-07945-3

**Published:** 2022-03-07

**Authors:** Mi Kyung Lee, Ocksim Kim, Kyoung-A. Kim, Sang Hui Chu

**Affiliations:** 1 Mo-Im Kim Nursing Research Institute, Yonsei University College of Nursing, Seoul, Republic of Korea; 2 Department of Nursing, Yonsei University College of Nursing and Brain Korea 21 FOUR Project, Seoul, Republic of Korea; 3 Department of Nursing, Yonsei University College of Nursing, 50-1, Yonsei-Ro, Seodaemun-gu, Seoul, 03722 Republic of Korea

**Keywords:** Trauma, Human behaviour

## Abstract

Refugees experience various kinds of trauma during the migration process, which can cause psychiatric problems such as posttraumatic stress disorder. However, in the process of overcoming traumatic experiences, they may also experience posttraumatic growth (PTG). This study examined the level of PTG and its associated factors among North Korean defectors, including posttraumatic stress symptoms (PTSS). In total, 212 North Korean defectors completed self-report questionnaires measuring PTG (PTG inventory), PTSS (Posttraumatic Stress Disorder Checklist, PCL-5), quality of life (WHOQOL-BREF), and various sociodemographic variables. Participants completed the survey online, from July 27 to August 4, 2020. Participants reported a moderate level of PTG scores (51.7 ± 15.4, range 0–80). To determine the impact of PTSS on PTG among North Korean defectors, we conducted a hierarchical multiple regression analysis. In the final model, several sociodemographic factors (years in South Korea, education in North Korea, religion, and employment status), overall quality of life (*β* = 0.321, *p* < 0.001), and PTSS (*β* = 0.162, *p* = 0.035) were positively associated with PTG, whereas living with family (*β* = − 0.1261, *p* = 0.040) and loneliness (*β* = − 0.401, *p* < 0.001) were negatively associated with PTG, accounting for 36.6% of the variance in PTG scores (*p* < 0.001). This is the first large-scale study describing the level of PTG and its associated factors among North Korean defectors residing in South Korea. Further, our study provides suggestions for future research in this area, and interventions for improving PTG among this group.

## Introduction

Trauma experienced in the course of violence, war, and migration can have adverse effects on the physical and mental health of survivors^1,2^. Common mental health challenges for trauma survivors include posttraumatic stress disorder (PTSD), anxiety, depression, and somatic and dissociative disorders^3,4^. However, in the process of overcoming trauma, there is also an opportunity for accelerated personal growth and realizing the value of freedom and human rights; this may include changes in self-awareness, interpersonal relationships, and philosophy of life^5,6^. Tedeschi and Calhoun define posttraumatic growth (PTG) as a “positive psychological change experienced as a result of the struggle with highly challenging life circumstances,” assuming it to be a positive and adaptive phenomenon, with greater adversity resulting in greater PTG^7,8^.

Refugees experience various kinds of trauma during the migration process, resulting in psychiatric problems, including an increased incidence of PTSD^1,9,10^. A large-scale meta-analysis, including samples of 64,000 refugees from 40 countries, estimated the overall prevalence of PTSD to be around 30%^3^. In the case of Korea, it is estimated that 33,000 North Korean defectors live in South Korea^11^. In the process of defection, these individuals experience various kinds of trauma and psychological stress, including separation from their families, threat to their lives, interpersonal violence, censorship by guards at the border, and anxiety caused by a sense of alienation in a foreign land^12^. In a study of 531 North Korean defectors, 81.4% reported experiencing trauma more than once; their traumatic experiences affected the prevalence of PTSD and depression^13^. The PTSD prevalence rate among North Korean defectors who entered South Korea within the last three years was 15.3%, about nine times higher than 1.7% among native South Koreans^14^. One study found that 53% of North Korean defectors who visited hospitals due to psychiatric problems were diagnosed with PTSD, and that those with PTSD exhibited a lower quality of life and had difficulty adapting to South Korean society. Difficulties in communicating getting along with South Koreans, and the feeling and experience of being ignored by native South Koreans affect the quality of life of North Korean defectors^15^. Moreover, posttraumatic stress symptoms (PTSS), such as flashbacks, nightmares, severe emotional distress, avoidance, and negative changes in thinking and mood, reduces the credibility of others, leading to difficulties in interpersonal relationships and negatively impacting quality of life^16–19^.

As PTSS and PTG in trauma survivors can have vastly different psychological consequences, North Korean defectors can experience both distress and growth through their hardships in North Korea, the process of entering South Korea, and even after their resettlement. While pathological phenomena such as PTSS and mental disorders among North Korean defectors have been the focus of considerable research, there has been less attention on PTG, which contributes to their overall growth and quality of life^5,20^. Moreover, clinicians and therapists working with traumatized North Korean defectors often focus on reducing PTSS, rather than facilitating positive change, despite the inherently problematic aspects of such a PTSD-centered approach^21^. As with any psychiatric diagnosis, PTSD-related stigma can make it harder for North Korean defectors to seek mental health services to reduce their PTSS. In addition, current trauma-focused treatments, such as cognitive-behavioral therapy or eye movement desensitization and reprocessing, involve higher costs, as they require trained clinicians and other resources.

Baumann et al.^21^ recently suggested that PTG as a phenomenon avoids the pitfalls of a PTSD-focused approach, and maintains mental health based on the conservation of resources model, which assumes that traumatized people seek to conserve the resources they have. According to this model, sustained or severe threat of loss of resources (e.g., adequate social support from family, social networks, or socioeconomic status) is critical to one’s well-being. While PTG occurs after a trauma, it develops not because of the traumatic event itself, but because of the individual’s efforts to overcome the traumatic experience through a new reality^8^. For example, Baumann et al.^21^ considered successful coping mechanisms used by displaced Syrian refugees as PTG; adapting to new norms, accepting changes, reaching out for support, and maintaining mental fortitude despite their loss of resources, led to the discovery of new resources. Similarly, North Korean defectors often lose critical resources in the process of escape, but they can recover from these traumatic experiences by finding new resources to buffer against possible future events. This process is associated with an increased appreciation of existing resources, more meaningful relationships, and a sense of personal strength following the traumatic and challenging events^21^.

Teododrescu et al.^9^ reported that PTG explained more of the variance in quality of life than PTSS in multi-traumatized psychiatric outpatients with a refugee background, suggesting a need to shift the treatment focus from reducing PTSS to increasing PTG, as well as improving their quality of life. However, the relationship between PTSS and PTG is still inconclusive. A positive relationship has been found in refugees^22^, cancer survivors^23^, and disaster survivors^24^, while a negative relationship has been reported in victims of interpersonal violence^25^. Thus, more research is needed to identify factors contributing to the successful acculturation of North Korean defectors in the South, and improve their quality of life through interventions based on empirical findings. As yet, no studies have examined the relationship between PTG and PTSS among North Korean defectors. This study aimed to explore PTG and its factors among North Korean defectors living in South Korea, through an examination of the relationship between PTSS and PTG, and identifying factors associated with PTG, considering sociodemographic factors as covariates.

## Method

### Participants

Participants were recruited through snowball sampling, with the assistance of key personnel in various online and offline communities for North Korean defectors, as well as centers providing psychological counseling to this population. The survey link was distributed through KakaoTalk (a smartphone messaging app) and text messages, and posted on online and offline bulletin boards by key personnel. The inclusion criteria were as follows: (1) North Korean defectors living in South Korea, (2) ages between 19 and 65 years, and (3) ability to understand the study aims and methods, and provide written informed consent. The exclusion criteria were as follows: (1) receiving psychiatric inpatient or outpatient treatment within the past six months, (2) currently receiving psychiatric medication, and (3) pregnant women. All participants completed self-report questionnaires online, from July 27 to August 4, 2020. After excluding incomplete surveys, a total of 212 valid surveys were analyzed (Table 1). Respondents’ mean age was 41.5 ± 11.9 years, 166 (78.3%) were women, and 170 (80.2%) had lived in South Korea for more than five years. The study was approved by the Institutional Review Board of Yonsei University Health Systems (Y-2020-0019) in South Korea and was conducted in accordance with relevant regulations and guidelines. Informed consent was obtained from all participants before completing the online survey.

**Table 1:** Participant demographic characteristics (n = 212).

	Mean ± SD or number	%
**Gender**		
Men	46	21.7
Women	166	78.3
Age (years)	41.5 ± 11.9	
**Education in North Korea**		
Elementary school	28	13.2
High school	126	59.4
College or higher	58	27.4
**Religion**		
Yes	113	53.3
No	99	46.7
**Years spent in South Korea**		
< 5	42	19.8
5 − 10	57	26.9
≥ 10	113	53.3
**Living with family**		
Yes	157	74.1
No	55	25.9
**Employment status**		
Employed	111	52.4
Not employed	101	47.6
Number of traumatic experiences	6.8 ± 3.7	
Loneliness	12.4 ± 4.2	
PTSS	29.6 ± 19.4	
PTG	51.7 ± 15.4	
Overall QoL	3.4 ± 1.0	
Overall health	3.2 ± 1.1	
Physical health	54.8 ± 21.3	
Psychological	62.3 ± 20.2	
Social relationships	57.2 ± 20.8	
Environment	56.9 ± 18.4	

^a^Data are presented as mean ± standard deviation or number (%).

^b^*PTSS* posttraumatic stress symptoms, *PTG* posttraumatic growth, *QoL* quality of life.

### Measurements

#### Sociodemographic characteristics

A questionnaire collected information about participants’ gender, age, education, marital status, religion, years in South Korea, living with family, employment, traumatic events experienced, and loneliness. Traumatic events were divided into five categories: disaster and accident-related; family-related; disease- and death-related; violence-related; and threat- and cultural shock-related trauma. A total of 16 types of traumatic events were listed^26^, and participants indicated experiencing and/or witnessing each type of event through “yes/no” responses. Loneliness was assessed using the Korean version of the UCLA Loneliness Scale (short-form; ULS-6)^27,28^, which is composed of six items: “I lack companionship”; “I feel part of a group of friends”; “I feel left out”; “I feel isolated from others”; “I am unhappy being so withdrawn” and “People are around me but not with me.” The items are rated on a four-point Likert scale, from “1” (not at all) to “4” (extremely), with total scores ranging from 1 to 24; higher values denote higher loneliness. The Cronbach’s alpha of this scale was 0.84.

#### Posttraumatic stress symptoms (PTSS)

The Posttraumatic Stress Disorder Checklist (PCL-5) is a 20-item self-report measure that assesses PTSD symptoms. It is commonly used to monitor symptom change during and after treatment, screening individuals for PTSD, and making a provisional PTSD diagnosis^29^. The PCL-5 is based on the Diagnostic and Statistical Manual of Mental Disorders Fifth Edition (DSM-5) criteria^30^. We used a Korean version of PCL-5^31^. It is a 20-item instrument that measures the severity of PTSS in the past month by asking “In the past month, how much were you been bothered by…”. The items are rated on a five-point Likert scale, from “0” (not at all) to “4” (extremely), with total scores ranging from 0 to 80; higher scores indicate a higher level of PTSS. The Cronbach’s alpha for this scale was 0.96.

#### Posttraumatic growth (PTG)

The Korean version of the PTG inventory is a 16-item self-report scale derived from the 21-item PTG inventory^7^. It contains four factors: changes in self-perception (six items), increase in interpersonal depth (five items), discovering new possibilities (three items), and increase in spiritual interest (two items). Five items were deleted in the process of validation of the Korean version due to cross-cultural differences^32^. For each item, respondents were given a statement describing a change they could have experienced (e.g., “I have developed new interests,” relating to changes in self-perception), and asked to indicate the degree to which they experienced this change as a result of experienced negative events. Items are rated on a six-point Likert scale, from “0” (not experienced) to “5” (very greatly experienced), with scores ranging from 0 to 80; higher scores indicate a greater positive change through coping with traumatic events. The Cronbach’s alpha for this scale was 0.93.

#### Quality of life

The World Health Organization Quality of Life Instrument, Short Form (WHOQOL-BREF) is a self-report instrument; Min et al.^33^ translated and developed the Korean version. This instrument comprises 26 items in four domains: physical health, psychological, social relationships, and environment^33^. Respondents indicate “how much,” “how completely,” “how good or how satisfied (they are),” or “how often” they experienced each item in the past two weeks (e.g., “How satisfied are you with your sleep?” relating to physical health). Responses are given on a five-point scale, ranging from “1” (not at all) to “5” (completely). We used the version where total scores are calculated on a scale of 0 to 100; higher scores indicate a better quality of life. Domain-specific Cronbach’s alpha values were: physical health = 0.86, psychological = 0.84, social relationships = 0.73, and environment = 0.84.

### Statistical analysis

Descriptive statistical analysis included frequencies and ratios or means and standard deviations for demographic variables. The normality of the distribution, linearity, and homoscedasticity for all continuous variables were confirmed by assessing histograms and scatter plots. To detect any differences in PTG scores according to categorical demographic characteristics, initial *t*-test or ANOVA were performed. Bivariate analyses were conducted to assess relationships between continuous variables, using Pearson’s correlation coefficient. To reduce the possibility of type 1 error, a more conservative *p-*value of 0.01 was used for multiple comparisons and bivariate correlations.

Multiple linear regression analyses were conducted to determine whether PTSS had a significant association with PTG after controlling for sociodemographic (gender, age, years in South Korea, education in North Korea, religion, living with family, employment, total number of trauma experience) and psychosocial variables. Hierarchical regression analyses were used to identify the individual contribution of each predictor to PTG after controlling for sociodemographic factors. The primary model (Model 1) consisted of sociodemographic factors (gender, age, years in South Korea, education on North Korea, religion, total number of trauma experience, living with family, employment). In the second model (Model 2), we added two psychosocial factors: loneliness and overall quality of life. In the final model (Model 3), PTSS was added to determine whether the level of PTSS predicted PTG. The variables were added sequentially in each step. Statistical tests were two-tailed, and p < 0.05 was considered statistically significant for multiple linear regression. All analyses were conducted using IBM SPSS Statistics 25 (IBM SPSS Inc, Armonk, New York, USA).

### Informed consent

Informed consent was obtained from all participants before completing the survey.

## Results

The survey responses clarified the details of participants’ trauma experiences. Family-related trauma was the most prevalent. From the total 212 respondents, 189 (89.2%) had been separated from their families; and 138 (65.1%) experienced death of family members. Other traumatic events were reported in the following order: verbal abuse and discrimination (60.4%), witnessing another person’s death (59.4%), life-threatening and extreme difficulties (50.9%), threat to own life (44.3%), domestic violence (41%), repatriation (39.6%), natural disaster (37.3%), divorce (35.4%), physical violence (33.0%), serious, life-threatening disease (31.1%), accidents (29.2%), torture (27.4%), sexual violence (25.0%), and parental divorce (15.6%).

Results related to PTSS, PTG, and quality of life are presented in Table 1. The mean PTSS total score was 29.6 (SD = 19.4); 43.9% participants scored above the 33-point cut-off score recommended by the National Center for PTSD^29^. The mean PTG total score was 51.7 (SD = 15.4), and domains had following means ± SD: changes in self-perception (21.4 ± 6.2), increase in interpersonal depth (15.2 ± 5.7), discovering new possibilities (10.0 ± 3.2), and increasing spiritual interest (5.1 ± 3.1). According to the quality of life results from the WHOQOL-BREF scale, 38.2% of the participants were satisfied with their general health, and 43.9% said they had a good quality of life. The WHOQOL-BREF domains had the following means ± SD: physical health (54.8 ± 21.3), psychological (62.3 ± 20.2), social relationships (57.2 ± 20.8) and environment (56.9 ± 18.4).

PTG scores were compared according to categorical demographic characteristic (Table 2). To reduce type 1 errors, a more conservative *p-*value of 0.01 were used for multiple comparisons and bivariate correlations, given the large number of comparisons made. PTG scores did not differ significantly between men and women (*p* = 0.110), according to educational level in North Korea (*p* = 0.075), years spent in South Korea (*p* = 0.336), and living with family (*p* = 0.244). However, PTG scores were significantly higher in those who were religious (p < 0.001) or were employed (*p* = 0.004). As shown in Table 3, the bivariate correlation analysis revealed significant positive relationships between PTG and overall quality of life (*r* = 0.471, *p* < 0.01), and medium to strong positive correlations with each domain of quality of life (*p* < 0.01), although it had a strong negative relationship with loneliness (*r* = − 0.442, *p* < 0.01). The negative correlation with PTSS and PTG was not statistically significant in the current study (*p* = 0.045). An inspection of the scatterplot of between PTG and PTSS showed a curvilinear relationship between PTG and PTSS, below the level of statistical significance (*p* = 0.017).

**Table 2:** PTG comparisons according to demographic characteristics among North Korean defectors (n = 212).

	PTG	p
**Gender**		0.110
Men	48.5 ± 14.6	
Women	52.6 ± 15.5	
**Education in North Korea**		0.075
Elementary school	46.8 ± 14.2	
High school	51.4 ± 16.1	
College or higher	54.7 ± 14.0	
**Religion**		< 0.001
Yes	55.1 ± 15.9	
No	47.7 ± 13.9	
**Years spent in South Korea**		0.336
< 5	48.7 ± 17.4	
5–10	51.5 ± 15.2	
≥ 10	52.8 ± 14.7	
**Living with family**		0.244
Yes	52.4 ± 15.5	
No	49.6 ± 15.1	
**Employment status**		0.004
Employed	52.5 ± 15.3	
Not employed	41.2 ± 13.3	

Data are presented as mean ± standard deviation.

*PTG* posttraumatic growth.

**Table 3:** Bivariate correlations among demographic variables, loneliness, PTG, PTSS, and QoL (n = 212).

	1	2	3	4	5	6	7	8	9	10	11
1. Age	1										
2. Loneliness	0.136*	1									
3. PTSS	0.181**	0.586**	1								
4. PTG	0.112	− 0.442**	− 0.138*	1							
5. Number of traumatic experiences	0.278**	0.191**	0.420**	0.087	1						
6. Overall QoL	0.001	− 0.445**	− 0.318**	0.471**	− 0.015	1					
7. Overall health	− 0.076	− 0.360**	− 0.392**	0.239**	− 0.064	0.444**	1				
8. Physical health	− 0.194**	− 0.470**	− 0.573**	0.207**	− 0.218**	0.437**	0.668**	1			
9. Psychological	− 0.038	0.634**	− 0.566**	0.507**	− 0.158*	0.615**	0.568**	0.712**	1		
10. Social relationships	− 0.136*	− 0.656**	− 0.409**	0.448**	− 0.188**	0.435**	0.483**	0.623**	0.700**	1	
11. Environment	− 0.076	− 0.510**	− 0.392**	0.416**	− 0.096	0.555**	0.452**	0.631**	0.710**	0.695**	1

Pearson’s correlation coefficients were used for the continuous variables.

*PTG* posttraumatic growth, *PTSS* posttraumatic stress symptoms, *QoL* quality of life.

*p < 0.05; **p < 0.01.

The relationship between PTSS and PTG was examined by identifying factors associated with PTG, including statistically significant variables (*p* < 0.01) in the univariate analyses (religion, employment, loneliness, and overall quality of life), and excluding the quality of life domains. We included additional variables (gender, age, education, year in South Korea, living with family, total number of traumatic experiences) based on a literature review. Table 4 shows the results of the hierarchical regression analysis. In Model 1, several sociodemographic factors (education in North Korea, religion, employment status) predicted PTG (*p* < 0.001). Loneliness (*β* = − 0.323, p < 0.001) negatively predicted PTG, while overall quality of life (*β* = 0.305, *p* < 0.001) positively predicted PTG in Model 2 (*p* < 0.001). Finally, PTSS severity was found to be a weak positive predictor of changes in PTG (*β* = 0.162, *p* < 0.035), accounting for an additional 1.4% of the variance in PTG scores (*p* < 0.001). The final models accounted for 36.6% of the PTG variance.

**Table 4:** Hierarchical multiple regression analyses for predicting PTG.

	Model 1			Model 2			Model 3		
	B	β	p	B	β	p	B	β	p
Gender	3.387	0.091	0.179	3.810	0.102	0.077	3.815	0.102	0.074
Age	− 0.005	− 0.004	0.962	0.037	0.028	0.661	0.043	0.034	0.601
**Years in South Korea (reference: < 5 years)**									
5–10 years	0.831	0.024	0.786	2.733	0.079	0.297	2.269	0.065	0.384
≥ 10 years	3.924	0.127	0.180	5.763	0.187	0.022	5.523	0.179	0.027
**Education in North Korea (reference: elementary)**									
High school	7.396	0.236	0.021	4.484	0.143	0.103	4.152	0.133	0.129
College or higher	10.062	0.292	0.007	7.661	0.222	0.016	6.706	0.195	0.034
Number of traumatic experiences	0.235	0.056	0.423	0.544	0.130	0.034	0.317	0.076	0.250
Religion	7.842	0.255	< 0.001	4.113	0.134	0.024	3.885	0.126	0.031
Living with family	0.730	0.021	0.764	− 4.091	− 0.117	0.058	− 4.412	− 0.126	0.040
Employment status	11.558	0.199	0.003	5.570	0.096	0.097	6.735	0.116	0.046
Loneliness				− 1.191	− 0.323	< 0.001	− 1.480	− 0.401	< 0.001
Overall QoL				4.904	0.305	< 0.001	5.167	0.321	< 0.001
PTSS							0.129	0.162	0.035
R square	0.156			0.392			0.405		
Adjusted R square	0.114			0.355			0.366		
R square change	0.156		< 0.001	0.236		< 0.001	0.014		< 0.001

Gender, years in South Korea, education in North Korea, religion, living with family, and employment were dummy coded. Gender: men = 0, women = 1, Religion: no = 0, yes = 1, Living with family: no = 0, yes = 1. Employment: no = 0, yes = 1.

*PTG* posttraumatic growth, *PTSS* posttraumatic stress symptoms, *QoL* quality of life.

## Discussion

This study investigated the relationship between PTSS and PTG among North Korean defectors residing in South Korea. To our best knowledge, this is the first study of the impact of PTSS on PTG among North Korean defectors. Further, our study provides insight into the challenges faced by those who work with traumatized North Korean defectors by identifying factors associated with PTG.

We found that North Korean defectors experienced diverse traumas in the process of defection, which is correlated with PTSS. As reported in previous studies, a higher number of traumatic experiences increase PTSD risk in North Korean refugees^34^. A higher PTSS level was associated with high loneliness and low quality of life, consistent with other studies on North Korean defectors. In a previous study, a group of North Korean defectors with PTSD reported a significantly lower quality of life than the non-PTSD group, and significantly higher difficulty in adapting to South Korean society^15^. In another study, PTSD symptoms such as anxiety, avoidance, and lethargy added to complex factors such as depression, resulting in poor receptivity to others and a negative impact on adaptation to South Korean society^35^.

However, in our study, a higher number of traumatic experiences was not associated with PTG, whereas PTSS severity was weakly associated with PTG among North Korean defectors. This could be related to the severity or type of trauma, or the accumulation of traumatic events over time, which are likely to play a role in PTG. The strength and linearity of the relationship between PTSS and PTG has been reported to differ by trauma type and age in a meta-analytic study^36^.

In the current study, an inspection of the scatterplot showed no evidence of a linear or curvilinear relationship between PTSS and PTG. The results of previous studies on the relationship between PTSS and PTG in refugees are controversial. A study on Tibetan refugees reported a positive correlation between PTG and PTSS^22^, whereas a study on Norwegian refugees reported a negative correlation between PTG and PTSD^9^. A review of PTG among refugees in Canada suggested that trauma exposure and PTG could have an inverse U-shaped relationship, with individuals who experienced a moderate level of trauma showing the highest level of growth^37^. In another study, the curvilinear relationship between trauma symptoms and growth suggested that there may be a range of challenging experiences that are sufficient to impel growth, but do not overwhelm or inhibit growth-promoting processes^38^. While PTG may be aligned with positive well-being for some people, for others, PTG may be a coping strategy to minimize distress, and may not be aligned with actual positive improvements in well-being.

In our study, the relationship between PTSS and PTG was statistically significant, but weak (*B* = 0.129, *p* = 0.035), which indicates that many other factors, along with PTSS, may play a role in differential levels of growth among North Korean defectors. In this study, years of residence in South Korea, education, religion, employment, and overall perception of quality of life were positively associated with PTG, whereas loneliness was negatively associated. However, contrary to our expectations, living with family was identified as a negative predictor of PTG. This is also inconsistent with previous qualitative findings that familial support promotes PTG^39^. It is possible that elevated levels of family conflict associated with differences in communication styles between generations or among couples, and difficulties in raising children during the resettlement period contributed to the lower PTG levels of refugees living with their families. Additional financial difficulties related to supporting a larger family may also have contributed to these results^40^. Further research is needed to explain this paradoxical phenomenon in this population.

The South Korean government provides early economic assistance and education on South Korean society to help North Korean defectors acculturate; these measures are aimed to move beyond temporary assistance and be of constant aid. Notably, in this study, the most frequently experienced traumatic events were family-related (e.g., separation from families and death of family members). Thus, to improve the PTG of North Korean defectors, appropriate interventions are needed to reduce their sense of loneliness by developing new social support systems. As reported in another study, psychological support from South Koreans was a positive predictor of PTG, and a small number of North Korean defectors reported overcoming traumatic experiences in anticipation of significant support from mainstream South Koreans^41^. Thus, the PTG of North Korean defectors can be increased by both restoring resources and lowering loneliness, with the help of public social support by South Koreans, especially those who are their initial point-of-contact during the early settlement period, such as personal protection officers or volunteers assisting North Korean defectors’ residential settlement at the Korea Hana Foundation, a non-profit public organization established by the Ministry of Unification. Therefore, it is necessary to strengthen the support competencies of first-contact South Korean personnel, facilitating their development as role models and mentors for North Korean defectors. In addition, professional counselors should be assigned to help and support North Korean defectors, assess positive changes, monitor their quality of life, and apply the medical approach for psychopathological symptom reduction.

According to a report on the impact of North Korean defectors’ traumatic experiences and social adjustment on health-related quality of life, social adjustment was the biggest factor affecting their quality of life, with an explanatory power of 30.1%^42^. A 12-week social support intervention for African refugees in Canada increased social integration, decreased social isolation, and expanded their coping repertoire. A major perceived benefit of the support program included connecting with people from the African refugees’ cultural communities^43^. Therefore, it was recently proposed that North Korean defectors with high levels of PTG be assigned as counselors, so that they can effectively help other North Korean defectors in the early settlement stage. They are expected to serve as effective role models for North Korean defectors who may feel avoidant toward South Korean counselors during the initial settlement process, and to provide practical strategies and know-how to help them overcome difficulties and achieve growth^5^. Successfully integrated positive psychological interventions, along with early economic assistance and adaptation education for South Korean society, beyond the mere treatment of mental illness, would promote various aspects of their quality of life, and facilitate connection to social networks and support.

Previous research found a relationship between higher levels of PTSS and greater physical health difficulties^44,45^. However, in contrast to our results, PTG made the strongest contribution to the physical, psychological, and environmental quality of life in multi-traumatized psychiatric outpatients with a refugee background in Norway^9^. These differences may be due to differences between study populations; in contrast to psychiatric outpatients, our participants were recruited from diverse online and offline communities of North Korean defectors, including centers providing them psychological counseling.

This study had some limitations. First, this was a cross-sectional study, which limits the interpretation of a causal relationship between PTSS and PTG. Longitudinal studies are required to explore causality, since PTG and may change depending on the period of North Korean defectors’ settlement in the South. Second, this study examined trauma experience based on 16 types of traumatic events; participants were asked to report whether or not they had experienced such events, but the timing, frequency, duration, and intensity of the trauma were unknown. It should be noted that these characteristics have been reported to influence both PTSS and PTG^36^. Third, the study participants were recruited from various online and offline communities, including centers that provide psychological counseling for North Korean defectors, using snowball sampling and an online survey. Thus, it is possible that estimates of PTSS may either be overrepresented, as the experiences of well-adjusted individuals may have been excluded, or underrepresented, as the experiences of those suffering from PTSD may have been excluded. Thus, any generalization of the findings must be approached with caution. Nevertheless, it is meaningful to study the relationship between PTSD and PTG among North Korean defectors in the context of socio-psychological aspects and their practical application in the field.

In conclusion, this was the first large-scale study describing the level of PTG and its associated factors, including PTSS, among North Korean defectors residing in South Korea. It provides insight into the challenges faced by North Korean defectors and shows how PTG can be improved by enhancing positive predictors and reducing negative factors. Our study also provides suggestions for future research in this area and interventions for improving PTG among this population.

## Data Availability

The datasets generated during and/or analyzed during the current study are available from the corresponding author on reasonable request.
